# Controlling Parkinson's Disease With Adaptive Deep Brain Stimulation

**DOI:** 10.3791/51403

**Published:** 2014-07-16

**Authors:** Simon Little, Alek Pogosyan, Spencer Neal, Ludvic Zrinzo, Marwan Hariz, Thomas Foltynie, Patricia Limousin, Peter Brown

**Affiliations:** ^1^Nuffield Department of Clinical Neurosciences, John Radcliffe Hospital, University of Oxford; ^2^Sobell Department of Motor Neuroscience & Movement Disorders, Unit of Functional Neurosurgery, UCL Institute of Neurology

**Keywords:** Medicine, Issue 89, Parkinson's, deep brain stimulation, adaptive, closed loop

## Abstract

Adaptive deep brain stimulation (aDBS) has the potential to improve the treatment of Parkinson's disease by optimizing stimulation in real time according to fluctuating disease and medication state. In the present realization of adaptive DBS we record and stimulate from the DBS electrodes implanted in the subthalamic nucleus of patients with Parkinson's disease in the early post-operative period. Local field potentials are analogue filtered between 3 and 47 Hz before being passed to a data acquisition unit where they are digitally filtered again around the patient specific beta peak, rectified and smoothed to give an online reading of the beta amplitude. A threshold for beta amplitude is set heuristically, which, if crossed, passes a trigger signal to the stimulator. The stimulator then ramps up stimulation to a pre-determined clinically effective voltage over 250 msec and continues to stimulate until the beta amplitude again falls down below threshold. Stimulation continues in this manner with brief episodes of ramped DBS during periods of heightened beta power.

Clinical efficacy is assessed after a minimum period of stabilization (5 min) through the unblinded and blinded video assessment of motor function using a selection of scores from the Unified Parkinson's Rating Scale (UPDRS). Recent work has demonstrated a reduction in power consumption with aDBS as well as an improvement in clinical scores compared to conventional DBS. Chronic aDBS could now be trialed in Parkinsonism.

**Figure:**
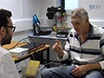


## Introduction

Parkinson's disease is a common severely disabling degenerative movement disorder for which long term medical treatment is unsatisfactory^1^. DBS is effective for advanced medically refractory PD but is limited in terms of efficacy, side effects and cost^2^. Conventional stimulation is set heuristically by a movement disorders specialist and left running continuously without change until the next clinic visit. Typical stimulation parameters are 60 msec pulse width, 3 V intensity, and 130 Hz repetition. However, this continuous high voltage stimulation may interfere with normal motor functioning^3^. Much as cardiac pacing has successfully transitioned from simple open loop systems to complex responsive closed loop systems, with an associated improvement in outcome, it is hoped that DBS can similarly be improved by making it responsive to underlying brain rhythms - adaptive DBS (aDBS)^4,5^.

In order to realize aDBS, it is firstly critical to identify reliable biomarkers of disease. Parkinson's disease is characterized by prominent beta (13-33 Hz) oscillations recorded throughout the basal ganglia circuit^2^. These beta oscillations are suppressed by levodopa and DBS in proportion to improvement in clinical state^6,7^. They are stable and robust in the long term and can be recorded from the same electrodes used for clinical DBS making them attractive targets for biomarkers^8^. In addition to beta oscillations, a range of other, more complex, potential biomarkers have been identified that have been shown to relate to severity of symptoms^2,9-12^.

Proof of principle of aDBS has now been demonstrated in a non - human primate model of PD^13^. This used a single cortical motor neuron to control stimulation with firing of the neuron triggering DBS stimulation after a fixed delay. The study reported that adaptive stimulation was more efficacious than conventional DBS. Recent work has since successfully extended aDBS to humans, the method for which is presented in this JoVE article^14^. This study investigated patients with PD in the immediate post-operative period prior to internalization of their wires and connection to their battery pack / stimulator. Beta oscillations were monitored in real time online and used to control the timing of high frequency stimulation. This led to a >50% reduction in power consumption and a 25% improvement in motor impairment compared to standard stimulation. These results will need to be replicated in the chronically implanted state where thresholds and effective stimulation parameters can change as well as drug levels. As such, biomarkers and control algorithms may need to be adjusted and matched accordingly and indeed may well require further complexity to adapt to this different parameter landscape. Devices that are equipped for longer term stimulation and recording are currently being developed and trialed in a research setting^15^. Meanwhile there is a need for a platform that will allow the possible benefits of adaptive DBS and the algorithms that underpin its performance to be further evaluated and refined. This is an important step, as errors and sub-optimal approaches are more difficult to reverse once systems are internalized for chronic use. Moreover, acute studies are necessary to motivate efforts in overcoming the challenges implicit in developing chronic internalized adaptive DBS system.

The goal of this methods report is to enable researchers to explore a range of different biomarkers and stimulation paradigms in DBS patients and to optimize parameters so as to maximize efficacy and minimize side effects / power consumption. It is the first method of its kind to be effective in patients with Parkinsonism and yet is relatively simple and easy to apply. The method is designed to investigate any DBS patient for whom there is a known LFP biomarker and who has a period of externalization post-operatively (up to 1 week period when electrode wires are extra-cranial and available for experimentation prior to battery/stimulator insertion).

## Protocol

### 1. Consent and Biomarker Identification

**This protocol has been reviewed and approved by the National Research Ethics Service Committee South Central - Oxford A.**

1. Identify appropriate subjects for study: subjects are those who are clinically identified as being suitable for deep brain stimulation (medically refractory Parkinson's disease). Note: Perform experiment after electrode implantation (day 1) and before battery/stimulator placement (day 7) whilst wires are externalized.
2. Consent patient for stimulation testing and overnight Parkinsonian medication withdrawal.
3. Following 12 hr withdrawal of Parkinsonian medication, connect externalized DBS electrodes to appropriate amplifier (amplifier must be designed, safety tested & validated for intracranial neurophysiological use in humans)^16^.
4. Record local field potential (LFP) at rest from all contacts bilaterally (L 0-3 & R 0-3.)
5. Create bipolar montages (0,2 and 1,3) by subtraction of recordings from adjacent contacts.
6. Perform power spectral analysis to identify patient specific beta peak frequency and bipolar contact pair with highest beta amplitude for stimulation testing. Note: majority of PD patients off medication have a beta peak^2^. If no beta peak is discernible off medication, the patient should be excluded from further analysis as this may represent DBS targeting complications.
7. Select contact for monopolar stimulation that is bridged by bipolar contact pair (0,2 or 1,3) with highest beta amplitude.

### 2. Connection of Patient to aDBS Setup

1. Connect the DBS electrode to the analogue amplifier and filter. Note: band pass filter between 3 - 37 Hz with x 9,100 gain.
2. Connect reference to patient. Note: Reference should be 5 cm x 5 cm neurostimulator gel electrode pad connected by a standard 2 mm monopolar electrode wire. Place the electrode over the left clavicle.
3. Connect analogue amplifier to A/D converter and portable computer running signal analysis software. Second stage digitally filter the signal around the patient specific beta peak with a 4 Hz pass band. Monitor beta amplitude and use this to control stimulation with a pre-set threshold. Smooth beta amplitude with a smoothing window (400 msec was heuristically effective in this setup.)
4. Connect D/A trigger to stimulator.
5. Connect stimulator to patient through amplifier. Note: For safety, ensure charge densities are limited to <30 μQ/cm^2^ and stimulator has linear input - output function. Optically isolate all connections to patient. Stimulator should be designed and tested with EN60601-1 medical safety standard as reference.

### 3. Testing of Conventional Continuous Stimulation

1. Turn on conventional high frequency stimulation, but initially at zero voltage (100 msec, 130 Hz, 0 V). Apply monopolar stimulation at contact bridged by bipolar contact pair with highest beta amplitude. Monitor stimulator readout continuously throughout experiment to ensure that stimulation delivered is as expected.
2. Slowly increase stimulation voltage by 0.5 V increments every few minutes, looking for clinical effect threshold. Establish clinically useful stimulation voltage (generally 1.5-3.0 V) accompanied by minimal or no side-effects such as paresthesia.

### 4. Testing of Threshold with on/off Switching and Trigger Setting

1. With the stimulation at the clinically effective voltage, switch the stimulator off with 250 msec ramping down.
2. With the stimulation at the clinically effective voltage, switch the stimulator on with 250 msec ramping up.
3. Enquire about paresthesia and any other possible side-effects experienced upon switching stimulation on and off.
4. If side effects are present, reduce the voltage by 0.25 V and repeat steps 4.1 to 4.3 until below paresthesia upon on/off switching threshold.
5. By titration find the threshold for paresthesia with on/off switching again and set the voltage just below (0.1 V) this threshold for further testing. If this is clinically ineffective then try other contact (choose contact 1 or 2). Note: switching stimulator on and off may also in some patients cause a spike artifact, possibly related to capacitance charging at the tissue electrode interface. As the signal was pass-band filtered in the beta band, before setting a trigger level, this artifact was greatly attenuated. Nevertheless, in a few instances this artifact was sufficient to cause self-triggering. To address this in these cases, we reduced the voltage of stimulation, and increased trigger threshold to escape self-triggering. Another way that this could be tackled in the future if necessary is through the incorporation of a lock out period after each trigger onset to avoid self-triggering.
6. Turn on stimulation at voltage just under the on/off switching paresthesia threshold determined above.
7. Increase beta amplitude trigger threshold to a level that results in the minimum time on stimulation whilst maintaining clinical effect. Aim for at least 50% reduction in time on stimulation.
8. Turn off stimulation and leave patient without stimulation (following set up and between test blocks) for 10 min to washout stimulation effect.

### 5. Testing of Patient Across Different Stimulation Conditions

1. Ensure blinding of patients to test conditions (adaptive DBS, conventional & off).
2. Apply each test condition for a minimum of a 5 min stabilization period with the condition running before clinical testing begins. Counterbalance test condition order across subjects. Fix the voltage, pulse width and stimulation frequency identically across test conditions so that the only difference relates to the timing of stimulation with regard to beta amplitude.
3. At a predetermined fixed point after commencement of stimulation (*e.g.*, as a minimum 300 sec), assess the patient clinically for effect through the UPDRS rating scale.
4. Video record assessment and then blind rate by independent experts offline (excluding rigidity.)
5. Perform clinical assessment using objective measures (accelerometry or actigraphy).

## Representative Results

Results using this method have recently been published using this protocol for beta amplitude (**Figure 1**)^14^. This study showed that clinically efficacious stimulation can be achieved despite a >50% reduction in time on stimulation (p < 0.001). It was also noted that time on stimulation tended to progressively drop despite a constant trigger threshold (2-tailed, 1-sample *t *test, t_7_ = 3.2, *p* = 0.01). Motor scores were improved by 66% and 50% during aDBS in the unblinded and blinded conditions, respectively. Despite the reduced amount of total stimulation - clinical outcome was 29% (p=0.03) and 27% (p=0.005) better in the aDBS group compared with the conventional DBS group (unblinded and blinded respectively, **Figure 2**).


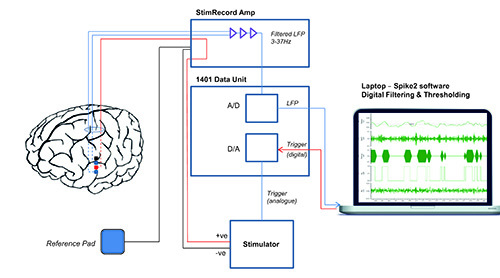
**Figure 1. Experimental setup.** Experimental setup for adaptive deep brain stimulation in externalized subjects. Bipolar local field potential (LFP) is passed through a custom built StimRecord amplifier that filters (3-37 Hz) and amplifies (x9,100). The analogue (A) output is passed to a data acquisition unit, which converts it to a digital (D) signal that is displayed on a portable computer. The signal is digitally filtered around the beta peak in real time and converted to beta amplitude by rectifying and smoothing. A threshold is set that triggers stimulation in a monopolar montage between the 2 bipolar recording electrodes when beta power crosses the threshold. Stimulation terminates when beta power drops again below threshold. *(Reprinted with permission from Annals of Neurology.)* Please click here to view a larger version of this figure.


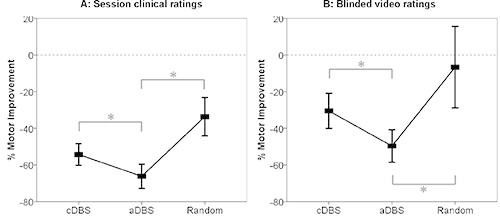
**Figure 2. Clinical improvements.** Mean ± SEM % change in hemibody UPDRS scores (items 20, 22, and 23) with different stimulation conditions as assessed unblinded during the experimental sessions (**A**) or from video recordings by blinded experts (**B**). Asterixes denote significant differences following correction for multiple comparisons by the false discovery rate procedure. All changes were significant from the unstimulated state, with the exception of the blinded score for random stimulation *(Reprinted with permission from Annals of Neurology.)* Please click here to view a larger version of this figure.

## Discussion

This article outlines a new method for the study and validation of adaptive deep brain stimulation in post-operative patients. DBS treatment is part of standard treatment for PD, essential tremor and dystonia and is being introduced and tested in a range of other conditions including cluster headache, epilepsy, Gilles de la Tourette syndrome, obsessive compulsive disorder and depression. At present, all clinical stimulation paradigms employ continuous, open-loop stimulation and although these simple stimulation paradigms are often effective, it is hoped that they can be significantly improved by making them responsive to disease appropriate biomarkers and thereby optimizing stimulation in an informed, patient specific manner. The method, outlined here, allows the testing of aDBS in patients externalized after their first surgery (electrode implantation), prior to internalization and implantation of the battery and stimulator. Using this method, it is hoped therefore that researchers can investigate the efficacy of aDBS using a range of biomarkers across the spectrum of disorders for which DBS is used. This may then lead to trials in the chronically implanted clinical setting.

The protocol that we have used and found to be successful is outlined above. We discerned a number of critical steps for fine-tuning the process in order to achieve successful aDBS. Parameters that can be controlled in this simple aDBS setup include voltage, trigger threshold, stimulation contact and ramping duration. These must all be balanced against the side effects of switching stimulation on and off (paresthesia), technical problems (recurrent ‘self' triggering) and clinical efficacy. Switching stimulation on and off causes a voltage dependent artifact in the LFP that, despite filtering, can potentially leak into the frequency range of interest. If this is severe, it can cause the system to self-trigger even in the absence of an elevation in the biomarker signal, -here beta activity in the local field potential. This does not represent a safety issue as it effectively results in the aDBS being on all the time and therefore mimicking cDBS which is known to be safe. However, it does result in a lack of reactivity to beta amplitude and the loss therefore of any potential benefit of aDBS over cDBS. We found that, if necessary, self-triggering could be avoided by reducing the stimulation voltage, raising the threshold or changing the stimulation contact. The 250 msec ramping of stimulation on and off was found to be satisfactory compromise with regard to preventing paresthesias whilst maintaining responsiveness of aDBS. At present parameters have to be adjusted heuristically in order to achieve the best response profile in individual patients and we have not yet identified consistent rules that are applicable at the group level to achieve this reliably. Nevertheless, in all patients studied thus far, we have found that heuristic adjustment of voltage, trigger threshold and stimulation contact enabled efficacious aDBS, and optimal parameters were identified in less than 30 min. It is hoped that the management of side effects (paresthesias from on/off switching) and artifact contamination (possibly related to tissue-electrode capacitance) can be further investigated and better understood to derive more generalized rules regarding their minimization.

The potential parameter space for exploration will also become larger and more complicated as the complexity of biomarkers and stimulation algorithms grow. For instance, high frequency power ratios, phase amplitude coupling and beta variability have all been shown to relate to Parkinsonian state^9,10,12,17^. The method described in this paper should enable the systematic investigation of such parameters and their effect on the clinical efficacy of stimulation in addition to their side effect profile. However, the thorough optimization of all parameters in the future is likely to be facilitated once DBS models and algorithmic optimization routines that focus on the response of the biomarker rather than clinical effects allow limitation of the parameter ranges to be searched empirically.

This method has demonstrated improved power consumption and clinical efficacy when compared to conventional DBS and has the potential to be further improved in PD with advancement in our understanding regarding biomarkers and stimulation patterning. In other conditions where DBS is used, much less is known regarding underlying pathophysiology and therefore corresponding biomarkers are yet to be fully determined. Significant further research is required to fully harness the potential of aDBS in Parkinsonism, and to explore its feasibility in a number of other potential neurological and neuropsychiatric conditions in which severity and impairment fluctuate over time.

## Disclosures

L.Z., M.H.: consultancy, travel support, speaking fees, Medtronic, St Jude. T.F.: consultancy, AbbVie Pharmaceuticals, St Jude, Medtronic; P.L.: consultancy, speaking fees, Medtronic, St Jude. P.B.: consultancy, Medtronic, Sapiens.

## References

[B0] Schrag A (2000). Dyskinesias and motor fluctuations in Parkinson's disease: A community-based study. Brain.

[B1] Little S, Brown P (2012). What brain signals are suitable for feedback control of deep brain stimulation in Parkinson's disease. Annals of the New York Academy of Sciences.

[B2] Chen CC, Brücke C (2006). Deep brain stimulation of the subthalamic nucleus: a two-edged sword. Current biology.

[B3] Modolo J, Legros A, Thomas AW, Beuter A (2011). Model-driven therapeutic treatment of neurological disorders: reshaping brain rhythms with neuromodulation. Interface Focus.

[B4] Priori A, Foffani G, Rossi L, Marceglia S (2012). Adaptive deep brain stimulation (aDBS) controlled by local field potential oscillations. Experimental neurology.

[B5] Kühn AA, Kupsch A, Schneider G, Brown P (2006). Reduction in subthalamic 8-35 Hz oscillatory activity correlates with clinical improvement in Parkinson's disease. The European journal of neuroscience.

[B6] Eusebio A, Cagnan H, Brown P (2012). Does suppression of oscillatory synchronisation mediate some of the therapeutic effects of DBS in patients with Parkinson's disease. Frontiers in integrative neuroscience.

[B7] Giannicola G, Rosa M (2012). Subthalamic local field potentials after seven-year deep brain stimulation in Parkinson's disease. Experimental neurology.

[B8] López-Azcárate J, Tainta M (2010). Coupling between beta and high-frequency activity in the human subthalamic nucleus may be a pathophysiological mechanism in Parkinson's disease. The Journal of Neuroscience.

[B9] Ozkurt TE, Butz M (2011). High frequency oscillations in the subthalamic nucleus: A neurophysiological marker of the motor state in Parkinson's disease. Experimental neurology.

[B10] Pogosyan A, Yoshida F (2010). Parkinsonian impairment correlates with spatially extensive subthalamic oscillatory synchronization. Neuroscience.

[B11] Chen CC, Hsu YT (2010). Complexity of subthalamic 13-35 Hz oscillatory activity directly correlates with clinical impairment in patients with Parkinson's disease. Experimental neurology.

[B12] Rosin B, Slovik M (2011). Closed-loop deep brain stimulation is superior in ameliorating parkinsonism. Neuron.

[B13] Little S, Pogosyan A (2013). Adaptive Deep Brain Stimulation in Advanced Parkinson Disease. Annals of neurology.

[B14] Afshar P, Khambhati A (2012). A translational platform for prototyping closed-loop neuromodulation systems. Frontiers in neural circuits.

[B15] Eusebio A, Thevathasan W (2011). Deep brain stimulation can suppress pathological synchronisation in parkinsonian patients. Journal of neurology, neurosurgery, and psychiatry.

[B16] Little S, Pogosyan A, Kuhn AA, Brown P (2012). Beta band stability over time correlates with Parkinsonian rigidity and bradykinesia. Experimental neurology.

